# Invadopodia play a role in prostate cancer progression

**DOI:** 10.1186/s12885-022-09424-4

**Published:** 2022-04-09

**Authors:** Valeria Manuelli, Fidelma Cahill, Harriet Wylie, Cheryl Gillett, Isabel Correa, Susanne Heck, Alex Rimmer, Anna Haire, Mieke Van Hemelrijck, Sarah Rudman, Claire M. Wells

**Affiliations:** 1 School of Cancer and Pharmaceutical Sciences, Kings College London, Rm.2.34A New Hunts House, London, SE1 1UL UK; 2 Department of Translational Oncology and Urology Research, Faculty of Life Sciences and Medicine, King’s College London, London, UK; 3 NIHR Biomedical Research Centre at Guy’s and St. Thomas’s Hospitals, King’s College London, London, UK; 4 Institute of Diabetes Research, Helmholtz Zentrum Munchen, Munich, Germany; 5 Department of Medical Oncology, Guy’s and St Thomas’ NHS Foundation Trust, London, UK

**Keywords:** Prostate cancer, Invadopodia, Circulating tumour cells

## Abstract

**Background:**

Invadopodia, actin-rich structures that release metallo-proteases at the interface with extra-cellular matrix, in a punctate manner are thought to be important drivers of tumour invasion. Invadopodia formation has been observed in-vitro and in-vivo in numerous metastatic cell lines derived from multiple tumour types. However, prostate cancer cell lines have not been routinely reported to generate invadopodia and the few instances have always required external stimulation.

**Methods:**

In this study, the invasive potential of primary prostate adenocarcinoma cell lines, which have never been fully characterised before, was investigated both in-vitro invadopodia assays and in-vivo zebrafish dissemination assay. Subsequently, circulating tumour cells from prostate cancer patients were isolated and tested in the invadopodia assay.

**Results:**

Retention of E-cadherin and N-cadherin expression indicated a transitional state of EMT progression, consistent with the idea of partial EMT that has been frequently observed in aggressive prostate cancer. All cell lines tested were capable of spontaneous invadopodia formation and possess a significant degradative ability in-vitro under basal conditions. These cell lines were invasive in-vivo and produced visible metastasis in the zebrafish dissemination assay. Importantly we have proceeded to demonstrate that circulating tumour cells isolated from prostate cancer patients exhibit invadopodia-like structures and degrade matrix with visible puncta. This work supports a role for invadopodia activity as one of the mechanisms of dissemination employed by prostate cancer cells.

**Conclusion:**

The combination of studies presented here provide clear evidence that invadopodia activity can play a role in prostate cancer progression.

**Supplementary Information:**

The online version contains supplementary material available at 10.1186/s12885-022-09424-4.

## Introduction

Prostate cancer (PCa) is the most commonly diagnosed cancer in man [1], with most PCa related deaths due to metastasis [2]. However, there are no efficient anti-metastatic drugs (migrastatics [3]) available to patients. Thus, there is a pressing need to better understand the molecular mechanisms that drive PCa invasion. It is widely believed that degradation of the extracellular matrix (ECM) is a key step in the metastastic process and can be achieved through the employment of actin rich membrane protrusions named invadopodia [4]. Invadopodia formation has been observed *in-vitro* in metastatic cell lines derived from multiple tumour types [5–8]. Importantly, there is also evidence of invadopodia driven invasion *in-vivo* [9–12]. However, it is not established whether PCa cells generate invadopodia structures nor whether they utilise invadopodia for matrix degradation. The “classical” PCa cell lines, such as DU-145 and PC3, although obtained directly from metastatic lesions, are not reported to spontaneously form invadopodia *in-vitro*. DU-145 cells can be driven to form invadopodia if triggered by peptide C-16 [13], LnCap cells exogenously expressing Tk5 (a known regulator of invadopodia activity) can exhibit invadopodia forming activity [14]. PC3 cells have been reported to degrade matrix [15] and can be stimulated to potentiate invadopodia with exposure to osteopontin/αvβ3 [16]. Thus, there is some preliminary evidence that invadopodia activity might hold physiological relevance in PCa progression.

In this study we investigated the invasive potential of primary prostate adenocarcinoma cell lines both *in-vitro* and *in-vivo*, exploring their ability to form invadopodia and to disseminate into distant sites using zebrafish embryo models. The zebrafish dissemination model is an established method of investigating cancer cell invasion though a complex tissue architecture [17, 18]. Furthermore, we established the presence of invadopodia activity in circulating tumour cells (CTCs) isolated from PCa patients. Taken together, these data strongly link invadopodia activity to PCa dissemination in both the experimental and clinical setting.

## Methods

### Cell culture

Human PCa cells 1532-CP2TX, 1535-CP1TX, and 1542-CP3TX [16] (referred to as CT-1532, -1535, -1542 cell lines) were cultured in Keratinocyte serum-free medium (KSFM) with 10% heat-inactivated fetal bovine serum, 1 mM penicillin–streptomycin, 25 μg/ml bovine pituitary extract (BPE), 5 ng/ml human EGF. Human pancreatic tumour cells AsPC-1 and PCa cells PC3 were cultured in RPMI-1640 media supplemented with 10% v/v HI-FBS and 1 mM penicillin–streptomycin.

### Growth assay

The cell viability was assessed by evaluating the [3-(4,5-dimethylthiazol-2-yl)-2,5-diphenyl-tetrazolium bromide] (MTT) reduction to formazan. Cells were seeded in triplicate in four 96-well plates at a density of 6 × 10^3^ per well. The media was removed, and cells were incubated 4 h in presence of 50 μl of MTT solution (2 mg/ml). Formazan crystals were dissolved by adding 50 μl of DMSO to each well, then absorbance was measured at 570 nm.

### Immunoblotting

Cell lysates were separated by acrylamide gel electrophoresis. Proteins were then transferred onto a nitrocellulose membrane followed by overnight incubation at 4 °C with the following primary antibodies: antimouse E-cadherin (Abcam, #ab1416), antimouse GAPDH (Merck, #MAB374). Membranes were washed in TBST and incubated with the respective HRP-conjugated secondary antibody (DAKO, #P0447). Proteins were visualised using Pierce enhance chemiluminescence (ECL) western blotting substrate and quantified by densitometric analysis using ImageJ software.

### Invadopodia assay

Coverslips were coated with a thin layer of Cy3-conjugated gelatine (EMD Millipore’s QCM Gelatin) according to the manufacturers protocol. Seeded cells were fixed in 4% paraformaldehyde (PFA) and stained.

### Immunostaining

*PFA* fixed cells on coverslips were permeabilised in 0.2% Triton X-100 and blocked with 5% BSA. Coverslips were incubated with the following primary antibodies antimouse E-cadherin (Abcam, #ab1416), antimouse Cortactin (Merck, #05–180), Alexa Fluor 647 anti-human CD45 (Biolegend, #304,020) followed by goat antimouse Alexa Fluor 488 IgG secondary antibody (Invitrogen, #A11001). Cells were imagined on an Olympus IX71 microscope.

### Zebrafish invasion assay

Zebrafish experiments were conducted under the UK Home Office project licence PPL 70/7912 and had been approved by the King’s College Ethical Review Committee. Approximately 500 GFP-tagged PCa cells or AsPC-1 cells were injected into the yolk sac using a Nikon SMZ-U zoom 1:10 Picospritzer II microinjection station. The embryos were checked at 24 h post injection to ensure that GFP signal was restricted to the yolk sac xenograft. 3 days post-injection the percentage of embryos with cancer cell tail invasion was calculated.

### CTCs isolation

Blood samples for CTC isolation were drawn from patients diagnosed with PCa with a Gleason score equal to 7 or more. For each patient, 7.5 ml of blood was collected in a 10 ml vacutainer EDTA tube, maintained at room temperature and processed within 4 h of collection. The study was conducted upon the obtainment of written informed consent and under the King’s Health Partners’ Prostate Cancer Biobank (KHP PCaBB) [39] blinding protocols for the protection of patients’ identities and sensitive data. The isolation of CTCs was achieved through Parsortix Cell Separation System (Angle Plc). GEN3 D6.5 cassettes and S99F programme were used following the manufacturer protocols. Isolated CTCs were immediately plated on Cy3-gelatin coated coverslips overnight. On the following day coverslips were incubated with Alexa Fluor 647 anti-human CD45 Antibody (Biolegend, #304,020) 30 min at RT before staining.

## Results

Routinely used PCa cell lines are not amenable to the study of spontaneous invadopodia formation. We sought to identify whether alternative PCa cell lines could be utilised. Three malignant cell lines, CT-1532, CT-1535 and CT-1542 had been previously isolated from radical prostatectomy specimens and immortalised using a recombinant retrovirus encoding the E6 and E7 transforming proteins of human papilloma virus serotype [19]. These three cell lines have been characterised previously as epithelial in origin and to exhibit a loss of heterozygosity on chromosome 8p, an associated feature of PCa [19, 20], the cells have been used to study prostate cancer progression [21–23]. We sought to investigate whether these cells could be used in an invadopodia assay.

### Prostate cancer cells lines represent an EMT transitionary phase

**Fig. 1:**
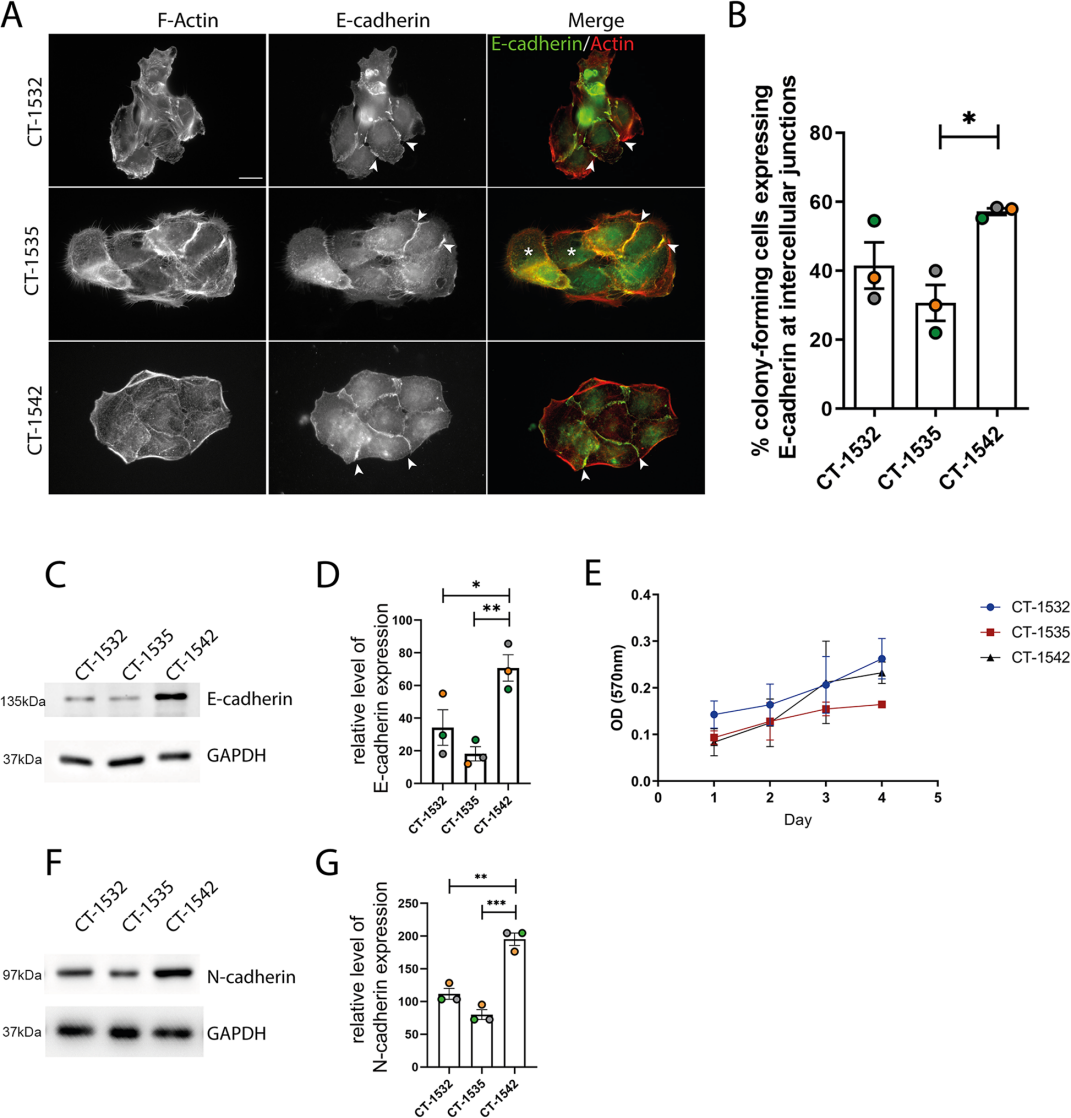
Differential cadherin expression levels. **A** cancer cell lines stained for E-cadherin (green) and F-actin (red). Scale bar = 10 μm. **B** Percentage of cells was forming colonies (cells forming adhesions with at least two neighbour cells) exhibiting E-cadherin signal at cell:cell junctions. Note that whilst in the 1542 cell colony all cells form at least one E-cadherin positive junction (arrowheads indicate examples of E-cadherin positive cell: cell junctions) the 1535 cell colony is less compact overall and contains cells that are not forming any E-cadherin positive cell: cell junctions (indicated by *) **C** Expression level of E-cadherin. **D** Quantification of E-cadherin expression by densitometric analysis corrected for the loading control (GAPDH) **E** Cell growth curve repeated over four consecutive days. **F** Expression level of N-cadherin **G** Quantification N-cadherin expression by densitometric analysis corrected for the loading control (GAPDH). Membranes were cut prior to hybridisation cropped Figure **C** and **F** are taken from three replicate analysis. Statistical significance was calculated with One-way Anova followed by Tukey’s multiple comparisons test, *p < 0.05, **p < 0.01, ***p < 0.005. All data is representative of 3 independent experiments mean ± SEM

Loss of E-cadherin has been linked to epithelial-mesenchymal transition (EMT) in PCa progression and metastasis [24]. However, it has been more recently recognised that EMT is not a binary switch but rather a series of transitionary states [25]. We therefore first sought to determine the E-cadherin status of our cell lines. All cell lines tested formed E-cadherin positive cell–cell junctions (Fig. 1A) with differential frequency (Fig. 1A, B). Interestingly total E-cadherin protein expression was significantly higher in CT-1542 cell line (Fig. 1C, D). Given the role of E-cadherin in regulation of cell proliferation [26], we tested proliferation rates, however no significant differences in growth curves were observed (Fig. 1E). To further explore the EMT transitionary status of these cell lines we tested for expression of N-cadherin [27]. Consistent with these cells being in a transitional state of EMT progression we detected expression of N-cadherin and the same differential of expression (Fig. 1F, G).

### Spontaneous invadopodia activity detected in prostate cancer cell lines

**Fig. 2:**
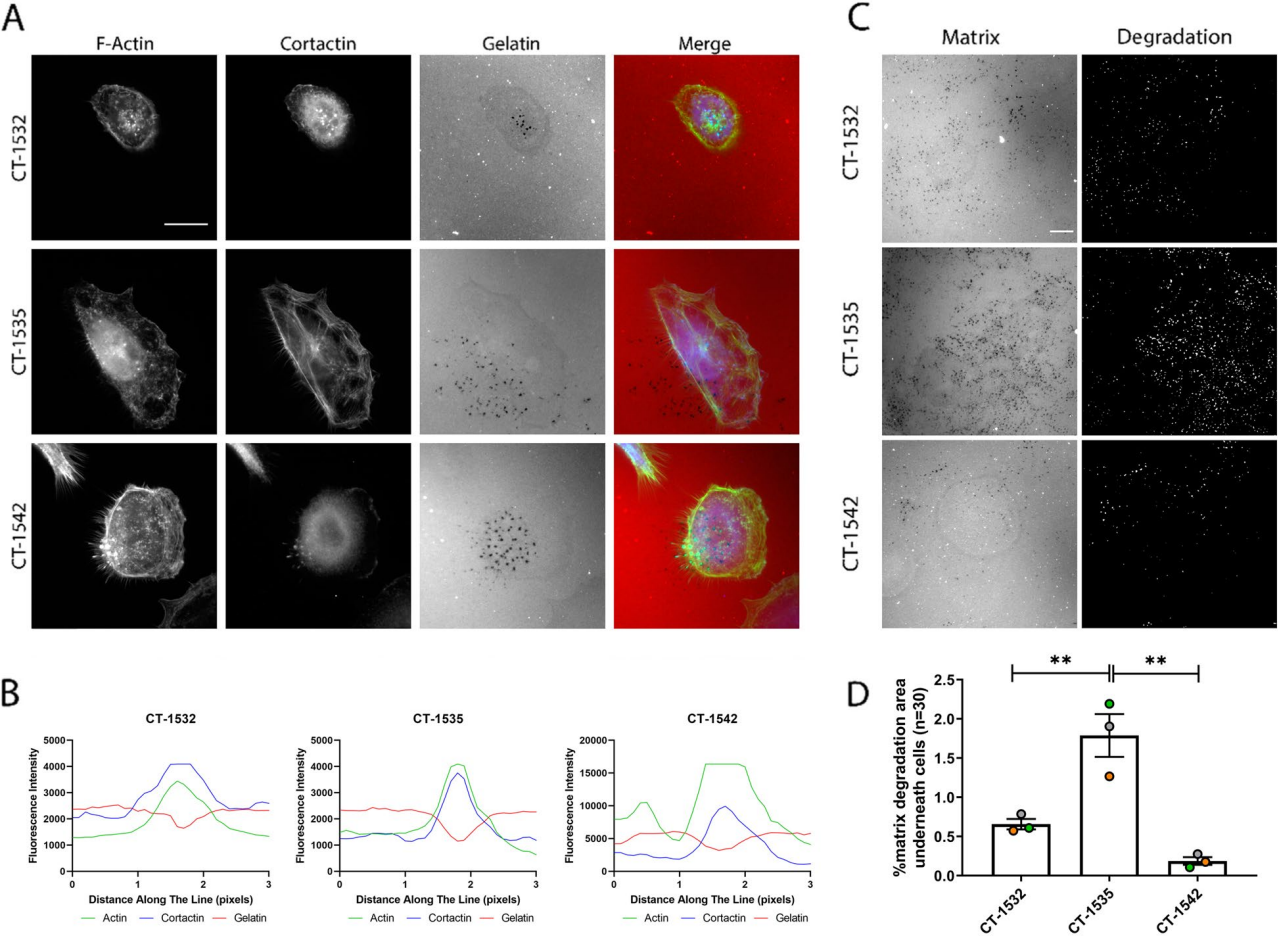
Primary prostate adenocarcinoma cell lines form invadopodia. **A** Cells were seeded on Cy3-conjugated gelatin for 24 h and stained for F-actin and Cortactin. **B** co-localisation of cortactin, F-actin and gelatin degradation. **C** Gelatin degradation. **D** percentage of degraded area underneath total cell area. *n* = 90 cells. All Data presented represent 3 independent experiments, mean ± S.E.M. Significance was calculated with One-way Anova followed by Tukey’s multiple comparisons test, **p < 0.005. Scale bar = 10 μm

PCa cell lines were subsequently tested for their ability to form invadopodia. Invadopodia were defined as actin-enriched puncta co-localizing with degraded matrix. Cells were additionally stained for cortactin a specific marker of invadopodia [28]. Remarkably, all the cancer cell lines screened showed invadopodia activity under basal conditions (Fig. 2A) with component colocalization confirmed (Fig. 2B). On the contrary as previously reported PC3 cells did not exhibit a notable capacity to synthetize invadopodia (Supplementary Fig. 1A, B). Quantitative measurement of the degraded area revealed that CT-1535 cells were comparatively the most invasive (Fig. 2C, D). As expected PC3 cells delivered an extremely low level of matrix degradation (Supplementary Fig. 1C).

### Confirmation of in-vivo invasion potential

**Fig. 3:**
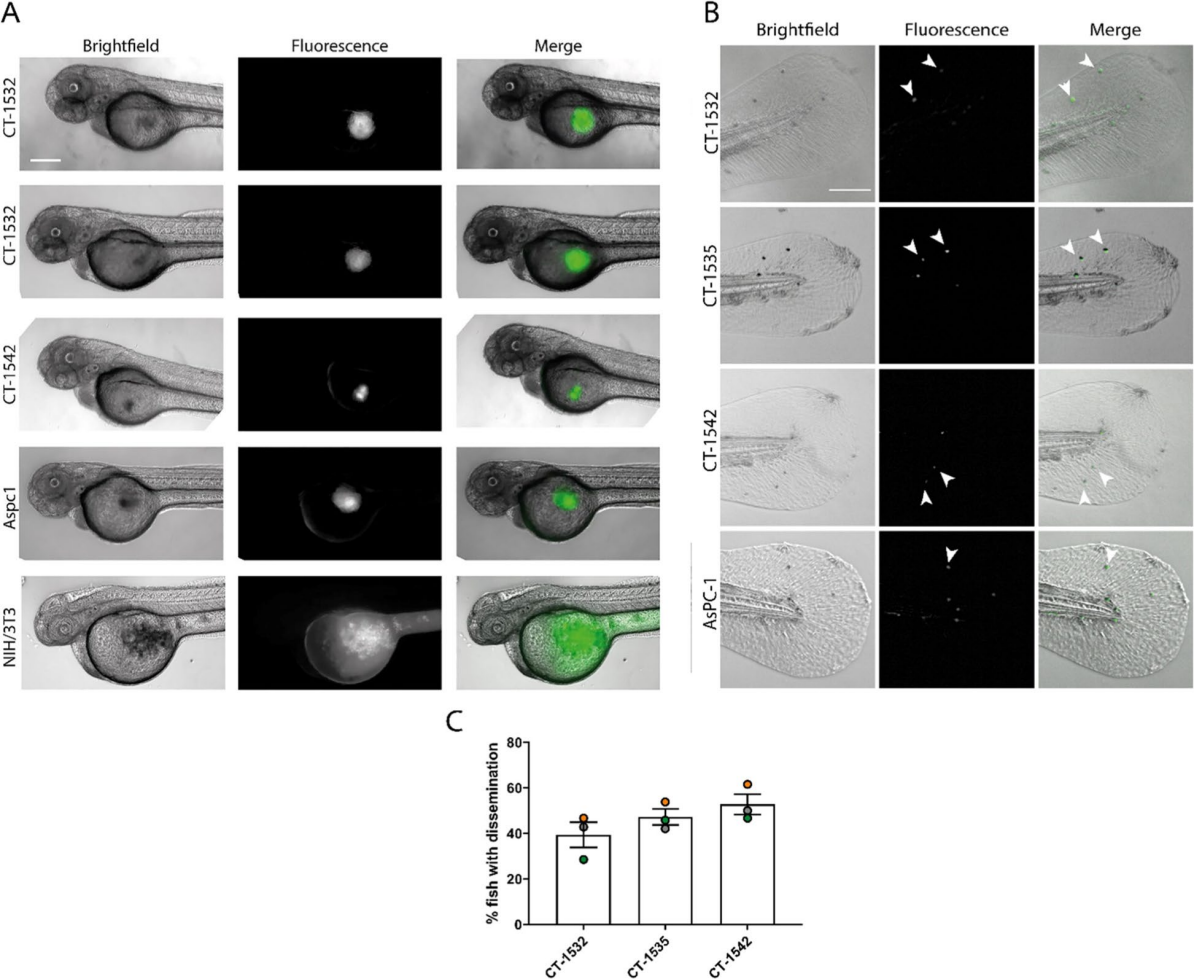
Primary prostate adenocarcinoma cell lines disseminate in-vivo. **A** xenograft formation in zebrafish yolk-sac invasion assay including an example of a non xenografting cell line NIH 3T3. **B** metastatic dissemination in the tail 3 days post injection, indicated by arrows. **C** Quantification of the percentage of embryos exhibiting metastasis in the tail region. Data are representative of three independent experiment, with at least 15 embryos screened for metastasis. Data are presented as mean ± S.E.M. Significance was calculated with One-way Anova followed by Tukey’s multiple comparisons test. Scale bar = 200 µm

Having evaluated the ability of our PCa cell lines to invade *in-vitro*, we tested their metastatic potential *in-vivo* using the zebrafish yolk sac invasion assay [29]. Fluorescently labelled (GFP) cells were injected into the zebrafish yolk sac and only embryos exhibiting a compact tumour mass (Supplementary Fig. 2A) with no GFP cells located outside the yolk sac were used in the assay. To confirm the validity of the assay, AsPC-1 cell line was used as positive control [29], while non tumorigenic mouse fibroblast NIH-3T3 cell line served as negative-control. All cell lines tested, except NIH-3T3, were able to form a compact xenograft in the yolk-sac of zebrafish embryos (Fig. 3A). Subsequent screening of the embryo distal tail region 3 days post injection (Fig. 3B) revealed the presence of cancer cell dissemination in at least 30% of embryos for all cancer cell lines (Fig. 3C and Supplementary Fig. 2B), demonstrating the *in-vivo* metastatic behaviour of the PCa cell lines.

### Circulating prostate cancer tumour cells have invadopodia activity

**Fig. 4:**
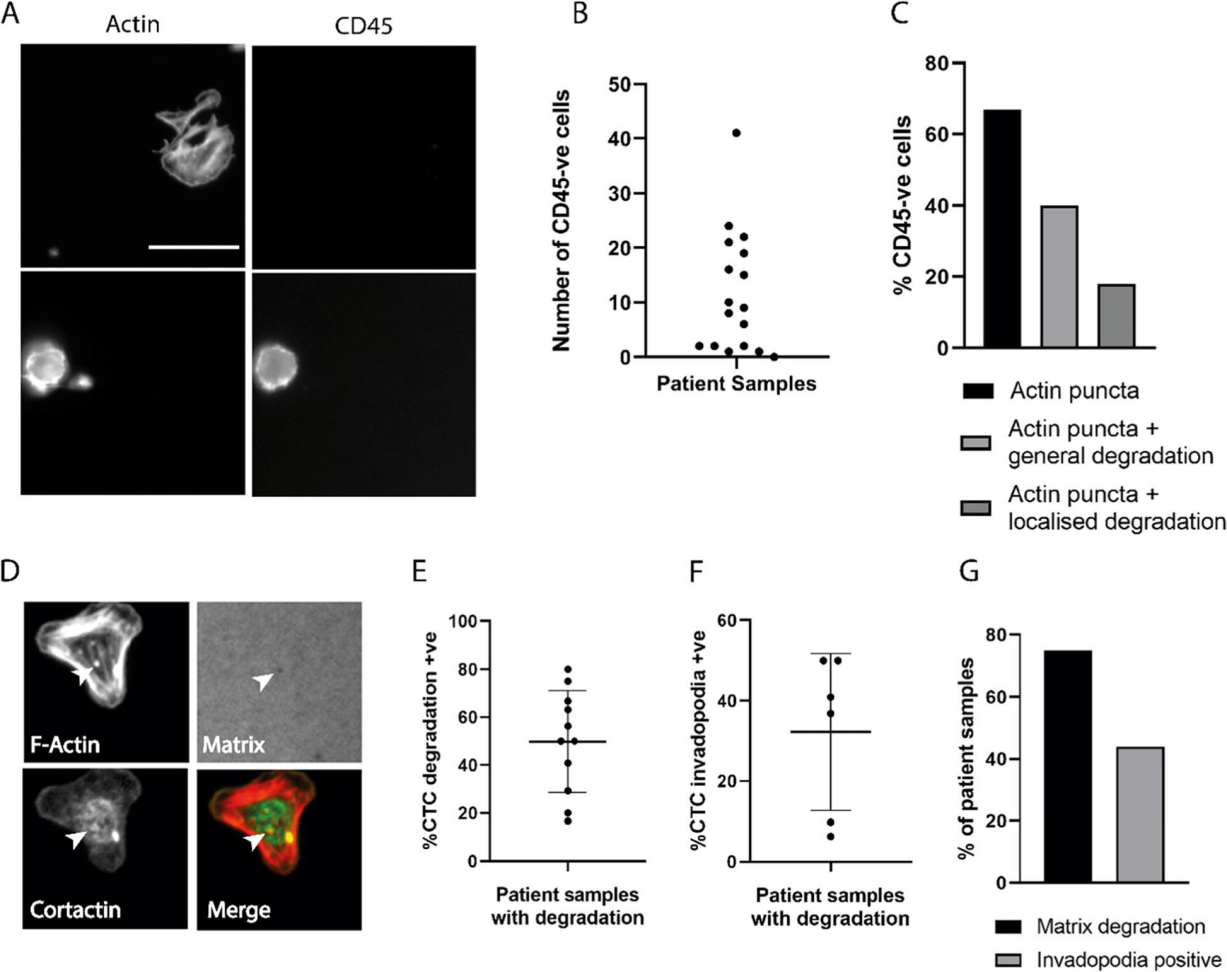
Circulating prostate tumour cells exhibit invadopodia activity. **A** circulating tumour cells (top) and hematopoietic cells (bottom) stained for CD45 and actin, imaged from the same field of view. **B** number of CD45-negative cells isolated from each blood sample. **C** percentage of CD45-negative cells displaying puncta and/or degradation. **D** PCa CTCs subjected to invadopodia assay and stained for F-Actin and Cortactin after 24 h incubation. Cells exhibited localised matrix degradation overlapping with actin puncta (white arrow) and cortactin puncta co-localising with actin (yellow arrow). **E** percentage of CTCs exhibiting matrix degradation properties. Samples without any visible degradation underneath CTCs were excluded. **F** percentage of CTCs exhibiting invadopodia formation in samples found positive for matrix degradation. Samples without any visible invadopodia activity were excluded. **G** percentage of samples with matrix degradation and invadopodia. Data are presented as Mean ± SD. Scale bar = 10 µm

Our findings support a role for invadopodia activity in the dissemination of PCa. To validate this hypothesis, we tested circulating tumour cells (CTCs) isolated from the peripheral blood of 17 PCa patients (minimum Gleason grade 7) (Supplementary table 1) for their ability to degrade the matrix in a punctate fashion. CTC cell preparations were stained for CD45 to exclude any haematopoietic cells from our evaluation. A cell stained for F-actin without CD45 staining is defined as a CTC (Fig. 4A, Supplementary Fig. 3A) [30]. From our patient cohort 16 of the 17 samples had at least one CD45 negative CTC with 8 patients delivering 10 or more CTC with the highest number recovered being 41 (Fig. 4B). Excitingly 67% of the CTCs analysed contained defined actin puncta, and more importantly 18% of CTC cells exhibited actin puncta colocalised with matrix degradation spots (Fig. 4C, Supplementary Fig. 3B). Indeed, specific staining of cells for cortactin revealed a bone fide invadopodia signal (Fig. 4D). Excluding those samples where the CTC cells did not degrade the matrix we detected that on average 50% of a patients CTCs were degradative at time of assay (Fig. 4E) although it was clear from the standard deviation that there is a considerable amount of patient variability. Moreover, where invadopodia activity was detected we found that on average 32% of a patients CTCs were positive for invadopodia (Fig. 4F); although again there is a considerable amount of patient variability. Overall, in totality 75% of patient samples displayed clear evidence of matrix degradation, while 40% of patient samples were positive for invadopodia activity in one or more CTC (Fig. 4G).

## Discussion

Invadopodia represents a promising therapeutic target to prevent cancer metastasis [31]. However, the contribution of invadopodia activity to PCa progression is uncertain, with no validated *in-vitro* study models. The data presented here suggest that human prostate autologous 1532, 1535 and 1542 cancer cell lines are a suitable research model for invadopodia studies in PCa.

Previous attempts to identify invadopodia activity in PCa cells have had limited success [16]. We hypothesise that this may be attributable to the use of cells derived from metastatic lesions which may have already downregulated this activity. It is interesting to note that in the cell lines tested here E-cadherin is retained at the cell surface and engaged in cell–cell junction formation. Indeed, studies conducted in melanoma demonstrated that cell lines with the most efficient degradative invasive activity were derived from a primary tumour [17]. Interestingly, the degradative ability exhibited an inverse relationship with E-cadherin levels and N-cadherin levels suggesting that the cell lines examined here represent a partial or transitory EMT state [25]. In this scenario metastatic cells do not completely lose E-cadherin expression but rather retain some E-cadherin expression and express mesenchymal markers such as N-cadherin. Indeed, recent research has suggested that retaining some E-cadherin expression supports metastatic dissemination [32]. Thus, our observations are consistent with the cell lines being representative of a more fluid EMT transitory state [25].

As the ability of cells to form invadopodia and degrade the matrix is thought to correlate with their invasive and metastatic capacity *in-vitro* and *in-vivo* [33], it is reasonable to hypothesise that the cell lines examined here hold metastatic potential. Indeed, all cancer cell lines performed optimally in the zebrafish dissemination assay. However, tail metastasis levels did not corelate with invadopodia activity, thus it is clear that there are multiple mechanisms employed by prostate cancer cells during invasion. It may be the case that cells *in-vivo* are able to switch to an alternative metalloproteases-independent mechanism of invasion that does not rely on invadopodia activity. PCa cells are reported to be capable of amoeboid cell migration [34, 35], a matrix degradation independent mode of migration [35, 36]. Alternatively, the higher level of E-cadherin expression in the CT1542 cells might support enhanced distal colonisation [37].

Circulating tumour cells can be thought to represent a snapshot of the cancer cells that have escaped the primary tumour. In the recent years, some evidence has emerged for an association between invadopodia and CTCs. CTCs isolated from breast cancer patients were able to generate invadopodia-like structures and invade into a 3D ECM [38]. We now find that PCa CTCs form invadopodia structures and are intrinsically capable of degrading the matrix; our novel finding paves the way for a new series of studies. Indeed, it is likely that given the longitudinal nature of our assay we have underestimated the level of invadopodia activity due to actin puncta turnover rates [39]. We speculate that PCa cells detaching from the primary tumour could employ invadopodia to navigate tissue and facilitate intravasation.

## Conclusions

Taken together our study supports further exploration of invadopodia activity in the PCa setting with the potential to develop novel therapeutics. Moreover, we propose that the PCa cell lines characterised here offer a robust and valid model for such future studies.

## Supplementary Information

**Additional file 1.** 

**Additional file 2.**

## Data Availability

The datasets used and/or analyzed during the current study available from the corresponding author on reasonable request.

## Abbreviations

PCa: Prostate cancer
ECM: Extracellular matrix
CTCs: Circulating tumour cells
BPE: Bovine pituitary extract
MTT: 3-(4,5-Dimethylthiazol-2-yl)-2,5-diphenyl-tetrazolium bromide
EMT: Epithelial-mesenchymal transition
