# Supplementary material for: Invadopodia play a role in prostate cancer progression
Source: BMC Cancer. 2022 Apr 9;22:386. doi: 10.1186/s12885-022-09424-4 (PMC8994910; doi:10.1186/s12885-022-09424-4)
Supplement: Supplementary file 1 — Additional file 1. [file 12885_2022_9424_MOESM1_ESM.pdf]

Raw data Figure 1C

Note the blots were cut prior to probing with E- cadherin and GAPDH - this is a view of the entire membranes

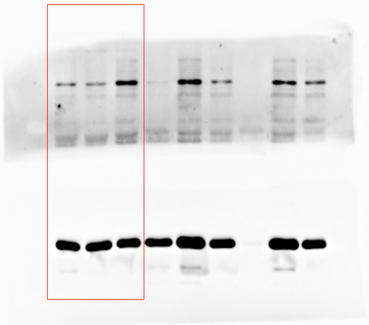

Raw data Figure 1E

Note the blots were cut prior to probing with N-cadherin and GAPDH - this is a view of the entire membranes

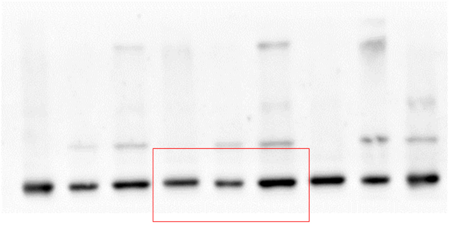

Ncadherin

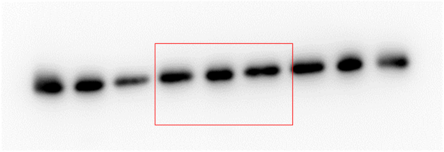

GAPDH
