# Supplementary material for: Invadopodia play a role in prostate cancer progression
Source: BMC Cancer. 2022 Apr 9;22:386. doi: 10.1186/s12885-022-09424-4 (PMC8994910; doi:10.1186/s12885-022-09424-4)
Supplement: Supplementary file 2 — Additional file 2. [file 12885_2022_9424_MOESM2_ESM.pdf]

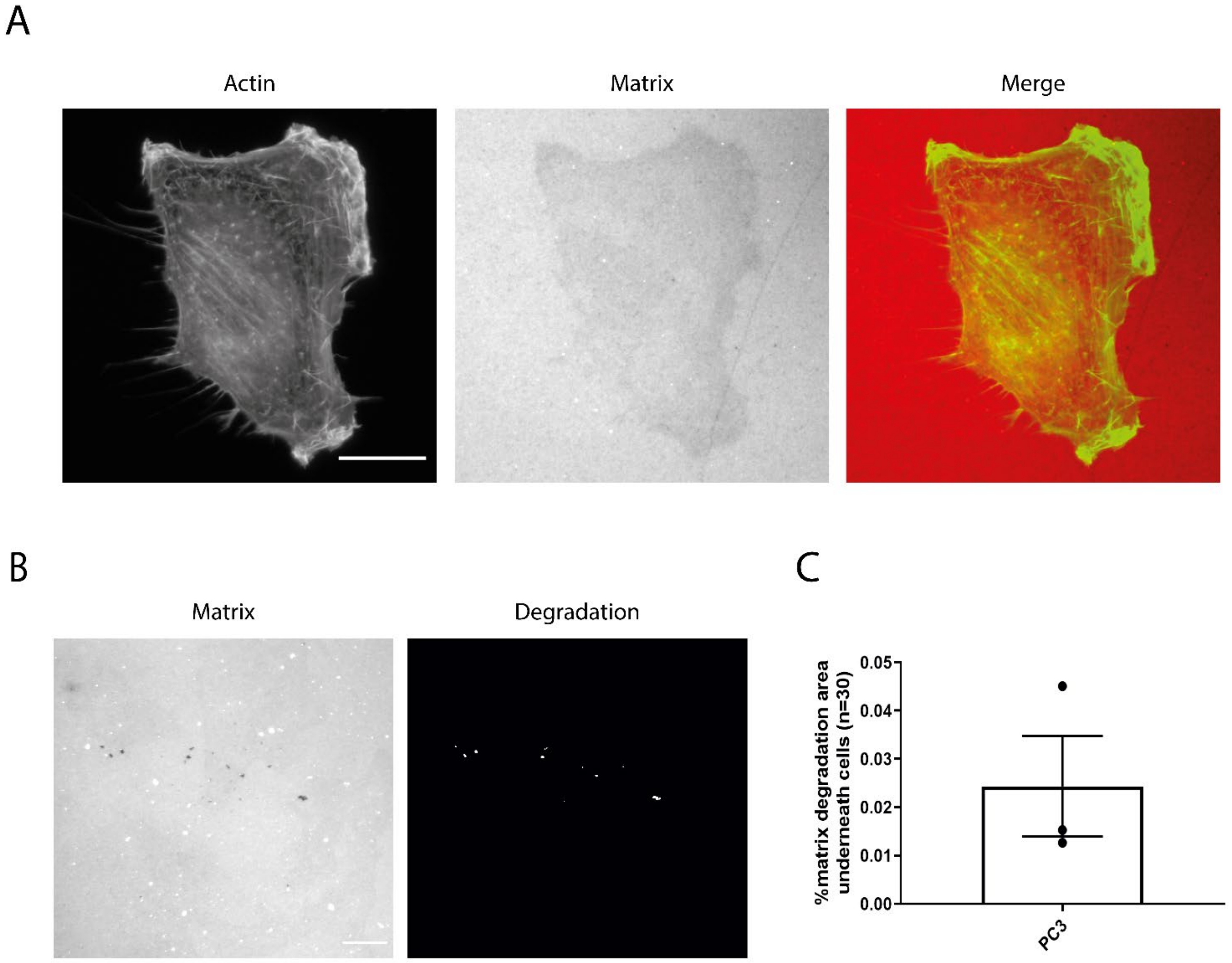

Supplementary Figure 1

(A) Representative invadopodia assay images of prostate cancer PC3 cell line. Cells were seeded on Cy3-conjugated gelatin for 24 hrs and stained for F-actin. No active invadopodia were identified. (B) Representative images of gelatin degradation reflecting the extent of the degradative ability of PC3 cell line. (C) Degradative ability was calculated as percentage of degraded area of the gelatin underneath total cells surface area corresponding to 30 cells over 3 independent experiments (n=3). Data are presented as mean values ± S.E.M. Scale bars = 10µm

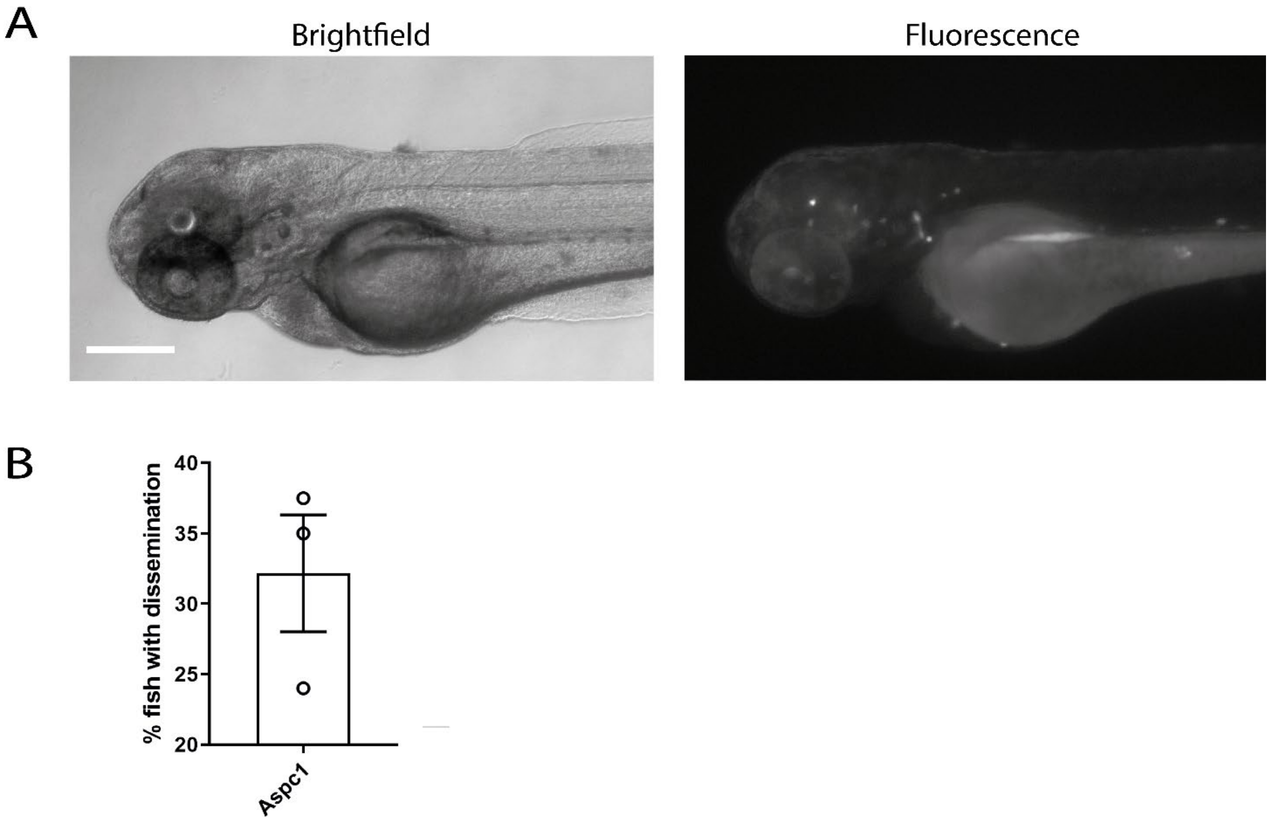

Supplementary Figure 2: Representative images of lack of xenograft formation in zebrafish yolk-sac invasion assay.

(A) GFP-labelled cancer cell lines were injected into 1dpf zebrafish embryos and screened for the presence of tumour mass into the yolk-sac. The image in A represents an embryo with a non-specific and disperse signal that was therefore excluded from the analysis. (B) Quantification of the percentage of embryos injected with AsPC-1 cells exhibiting metastasis in the tail region. Data are representative of three independent experiment, with at least 15 embryos screened for metastasis at the end of each experiment. Data are presented as mean values ± S.E.M. Scale bar=200μm.

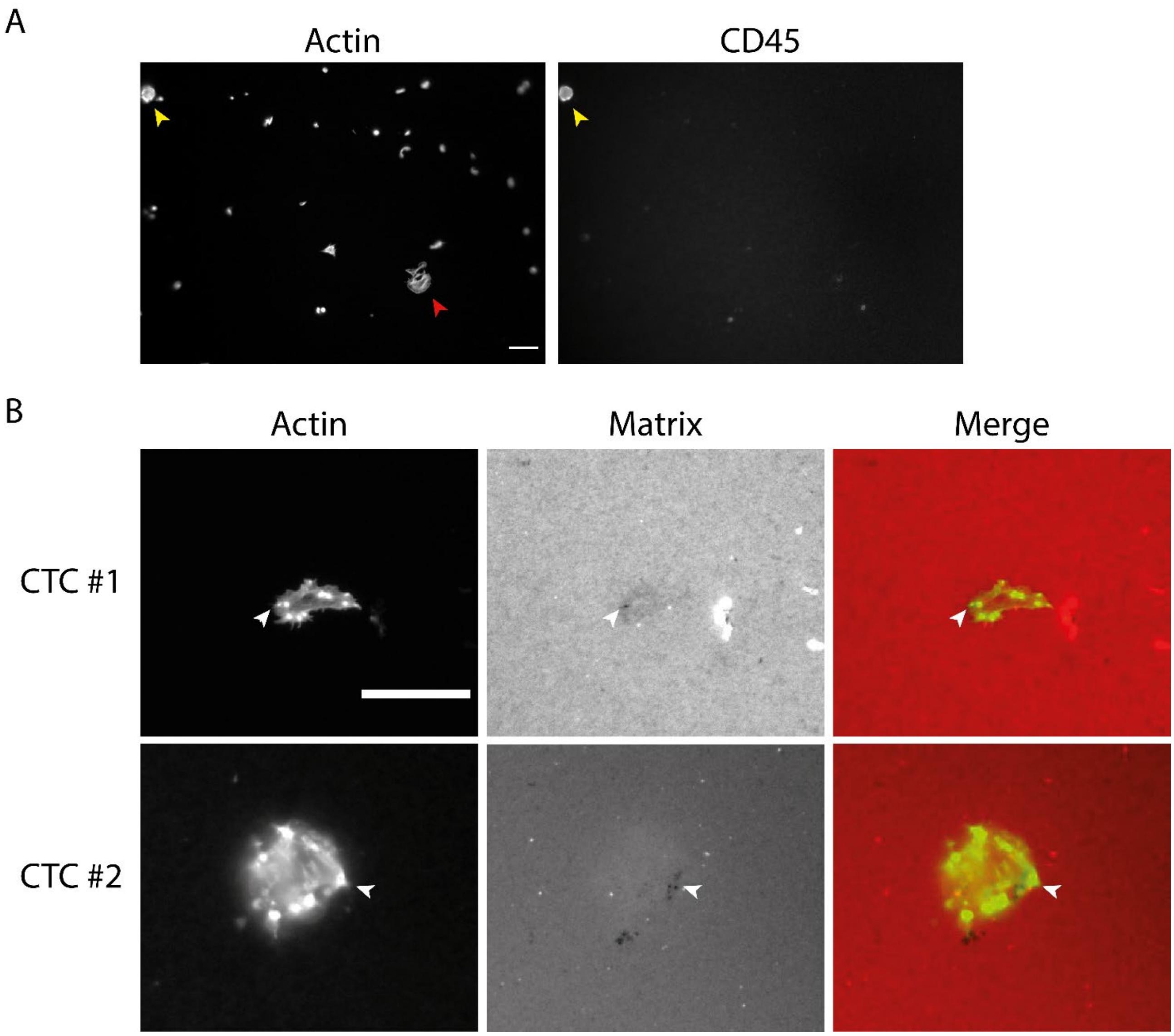

Supplementary Figure 3: Representative images of human prostate CTCs

(A) Representative images of circulating tumour cell (red arrow) and hematopoietic cell (yellow arrow) isolated via Parsortix System from PCa blood samples and stained for the surface antigen CD45 and imaged from the same field of view. The circulating tumour cell can be clearly distinguished by the absence of CD45 signal, which is expressed by the hematopoietic cell depicted together. (B) Additional images of isolated circulating tumour cells subjected to invadopodia assay and exhibiting degradation underneath the cell's surface area overlapping with actin-rich puncta. Scale bar=10µm.

CTCs					
Patient	**Gleason score**	**# of isolated CTC**	**Actin puncta**	**Degradation under cell**	**Puncta aligned with degradation**
1	4+5	1	0	0	0
2	Unknown (Diagnosed Radiologically)	2	0	0	0
3	4+5	24	15	12	**12**
4	5+5	0	0	0	0
5	4+5	2	1	1	**1**
6	5+4	9	6	6	0
7	4+4	10	1	8	0
8	Unknown (Diagnosed Radiologically)	1	0	0	0
9	4+5	8	5	6	0
10	4+5	22	19	9	**9**
11	3+4	16	12	9	**1**
12	4+5	21	15	8	0
13	3+4	6	3	1	0
14	4+3	15	9	3	**1**
15	3+4 at diagnosis, later upgraded to 4+5	41	31	21	**4**
16	4+5	2	1	0	0
17	4+5	19	15	12	**7**

Supplementary Table 1: Patients' clinical data and outcome of invadopodia assay performed with human prostate CTCs.
